# Inhibition of voltage-gated Na^+^ currents by eleclazine in rat atrial and ventricular myocytes

**DOI:** 10.1016/j.hroo.2020.05.006

**Published:** 2020-05-25

**Authors:** Rachel E. Caves, Alexander Carpenter, Stéphanie C. Choisy, Ben Clennell, Hongwei Cheng, Cameron McNiff, Brendan Mann, James T. Milnes, Jules C. Hancox, Andrew F. James

**Affiliations:** ∗ School of Physiology, Pharmacology & Neuroscience, University of Bristol, Bristol, United Kingdom; † Xention Ltd, Cambridge, United Kingdom

**Keywords:** Antiarrhythmic drug, Atrial myocytes, Cardiac regional heterogeneity, *I*_Na_, *I*_Na,Late_, Na^+^ channel blocker, Ventricular myocytes

## Abstract

**Background:**

Atrial-ventricular differences in voltage-gated Na^+^ currents might be exploited for atrial-selective antiarrhythmic drug action for the suppression of atrial fibrillation without risk of ventricular tachyarrhythmia. Eleclazine (GS-6615) is a putative antiarrhythmic drug with properties similar to the prototypical atrial-selective Na^+^ channel blocker ranolazine that has been shown to be safe and well tolerated in patients.

**Objective:**

The present study investigated atrial-ventricular differences in the biophysical properties and inhibition by eleclazine of voltage-gated Na^+^ currents.

**Methods:**

The fast and late components of whole-cell voltage-gated Na^+^ currents (respectively, *I*_Na_ and *I*_NaL_) were recorded at room temperature (∼22°C) from rat isolated atrial and ventricular myocytes.

**Results:**

Atrial *I*_Na_ activated at command potentials ∼5.5 mV more negative and inactivated at conditioning potentials ∼7 mV more negative than ventricular *I*_Na_. There was no difference between atrial and ventricular myocytes in the eleclazine inhibition of *I*_NaL_ activated by 3 nM ATX-II (IC_50_s ∼200 nM). Eleclazine (10 μM) inhibited *I*_Na_ in atrial and ventricular myocytes in a use-dependent manner consistent with preferential activated state block. Eleclazine produced voltage-dependent instantaneous inhibition in atrial and ventricular myocytes; it caused a negative shift in voltage of half-maximal inactivation and slowed the recovery of *I*_Na_ from inactivation in both cell types.

**Conclusions:**

Differences exist between rat atrial and ventricular myocytes in the biophysical properties of *I*_Na_. The more negative voltage dependence of *I*_Na_ activation/inactivation in atrial myocytes underlies differences between the 2 cell types in the voltage dependence of instantaneous inhibition by eleclazine. Eleclazine warrants further investigation as an atrial-selective antiarrhythmic drug.

**Key Findings**

▪ Differences exist between rat atrial and ventricular myocytes in the voltage dependence of activation and inactivation of fast Na^+^ current (*I*_Na_).
▪ Eleclazine blocks *I*_Na_ in a use-dependent manner consistent with preferential association with activated states of the channel in both atrial and ventricular myocytes.
▪ Eleclazine shows unusually rapid dissociation from the sodium channel in both atrial and ventricular myocytes.
▪ The study shows an atrial-selective instantaneous inhibition of *I*_Na_ by eleclazine.
▪ Eleclazine partially reverses the shortening of atrial effective refractory period induced by the muscarinic agonist carbachol.

## Introduction

Atrial fibrillation (AF) is characterized by a rapid and irregular electrical activation of the atria and is associated with significant morbidity and mortality, principally through an elevated risk of thromboembolism and ischemic stroke.^1^ AF is the most common clinical arrhythmia and its prevalence can be expected to rise with aging of the population, with consequent increase in socioeconomic burden of the disease.^2^ The elevated atrial rate during AF causes electrical and structural remodeling that stabilizes the arrhythmia, establishing a progressive nature to the condition.^1^ Effective early intervention to prevent and/or control the arrhythmia is therefore desirable.^1^

As activation of voltage-gated Na^+^ channels underlies the propagation of the cardiac action potential, and their subsequent inactivation initiates a refractory period, Na^+^ channels represent an important target for antiarrhythmic drugs: the combined effects of reduction in membrane excitability, conduction velocity (CV) slowing, and prolongation of refractory period resulting from Na^+^ channel block can both suppress triggered activity and extinguish re-entrant circuits.^3^ Na^+^ channel blockers with relatively slow dissociation kinetics are effective in the cardioversion of early-onset AF and the maintenance of sinus rhythm.^1^^,^^4^ Notably, the class Ic antiarrhythmic drugs flecainide and propafenone are recommended as a suitable “pill-in-the-pocket.”^1^^,^^5^

However, despite the effectiveness of class Ic antiarrhythmic drugs in the treatment of AF, the Cardiac Arrhythmia Suppression Trial showed that these drugs carried an increased mortality in patients with myocardial infarction, precluding their use in such individuals.^6^ As a result, there has been considerable interest in alternative agents that allow atrial-selective targeting of voltage-gated Na^+^ channels.^7^^,^^8^ In principle, atrial selectivity of action might arise through (1) atrial-ventricular differences in the molecular, biophysical, or pharmacological properties of voltage-gated Na^+^ channels and/or (2) atrial-ventricular differences in the resting membrane potential and action potential configuration. Compared with the voltage-gated Na^+^ channel current (*I*_Na_) of ventricular myocytes, atrial *I*_Na_ inactivates and activates at more negative potentials, with more rapid onset, and recovers more slowly from inactivation.^7^^,^^9^, ^10^, ^11^, ^12^

The antianginal agent ranolazine (Ranexa) is a prototypic example of a drug with atrial-selective action against voltage-gated Na^+^ channels.^7^ Ranolazine shows use- and voltage-dependent block of voltage-gated Na^+^ channels through binding to the local anesthetic binding site within the Na^+^ channel vestibule and is relatively selective for the late Na^+^ current (*I*_NaL_).^13^, ^14^, ^15^, ^16^ The atrial-selective action of ranolazine against Na^+^ channels arises through a preferential block of the activated state and trapping of the drug in the inactivated state in combination with atrial-ventricular differences in (1) the voltage dependence of *I*_Na_ activation and inactivation and (2) resting membrane potential and diastolic interval (DI).^7^^,^^12^^,^^16^ Ranolazine suppresses the incidence of AF in anginal patients and is suggested to be effective in pharmacological cardioversion of patients with early-onset AF and the prevention of postoperative AF.^17^, ^18^, ^19^, ^20^ However, at therapeutically relevant concentrations, ranolazine also inhibits the rapid delayed rectifier current (*I*_Kr_) and delays ventricular repolarization.^21^ As use of ranolazine has been associated with cases of torsades de pointes arrhythmia,^22^^,^^23^ albeit rarely, an atrial-selective antiarrhythmic without effect on *I*_Kr_ is desirable. It can be anticipated that selective inhibitors of *I*_NaL_ that show preferential block of the activated state with rapid rates of association and dissociation would also show an atrial-selective inhibition of the fast component of *I*_Na_ at higher rates.^15^^,^^16^ For example, the triazolopyridine GS-967 (now known as PRAX-330), a proof-of-concept selective *I*_NaL_ inhibitor with little activity against *I*_Kr_, has been shown to have an atrial-selective action against action potential duration, postrepolarization refractoriness, and the maximum upstroke velocity of the action potential, although whether the atrial-selective action of the drug extends to fast *I*_Na_ itself remains unclear.^24^^,^^25^ However, the low therapeutic index of GS-967 associated with nonselective effects on a range of neuronal Na^+^ channel isoforms and high brain penetrance make it unattractive for development as an antiarrhythmic drug.^24^

In contrast, eleclazine (ELE; formerly GS-6615) is a selective *I*_NaL_ blocker with properties similar to ranolazine that has been reported to be safe and well tolerated and to shorten the QT_cF_ interval in patients with long QT3 syndrome.^26^^,^^27^ ELE has been shown to inhibit fast *I*_Na_ in a use-dependent manner consistent with preferential activated state block with little effect on *I*_Kr_^27^^,^^28^ and to reduce spontaneous AF in an intact porcine model.^29^ The objectives of this study were to investigate atrial-ventricular differences in the properties of *I*_Na_ and its inhibition by ELE in rat cardiac myocytes.

## Methods

Detailed methods are available in supplemental information online.

### Rat cardiac myocyte isolation

Rat left ventricular and left atrial myocytes were isolated as described previously using procedures approved by the University of Bristol Animal Welfare and Ethics Board in accordance with UK legislation and the *Guide for the Care and Use of Laboratory Animals*.^30^^,^^31^

### Whole-cell recording of voltage-gated Na^+^ currents

Whole-cell Na^+^ currents were recorded at room temperature using the patch clamp technique. The fast component of the Na^+^ current (*I*_Na_) was recorded using symmetrical internal and external [Na^+^] (5 mM) whereas the late Na^+^ current (*I*_NaL_) was recorded using 70 mM external and 5 mM internal [Na^+^] (Supplemental Table 1).

### Electrophysiological recordings from whole hearts

Atrial effective refractory period (ERP) and CV were measured in Langendorff-perfused rat hearts as described previously.^32^

### Eleclazine

ELE (3,4-dihydrobenz-[1,4]oxazepin-5(2H)-one) was a gift of Dr James T. Milnes (Xention Ltd, Cambridge, UK). ELE was used at 10 μM to produce use-dependent inhibition of fast *I*_Na_.^27^^,^^28^

### Statistics

Data are presented as the mean ± standard error of the mean. The limit of statistical confidence was *P* < .05. Curve fitting was performed by nonlinear least squares using Igor Pro v6 (Wavemetrics Inc, Tigard, OR).

## Results

**Figure 1:**
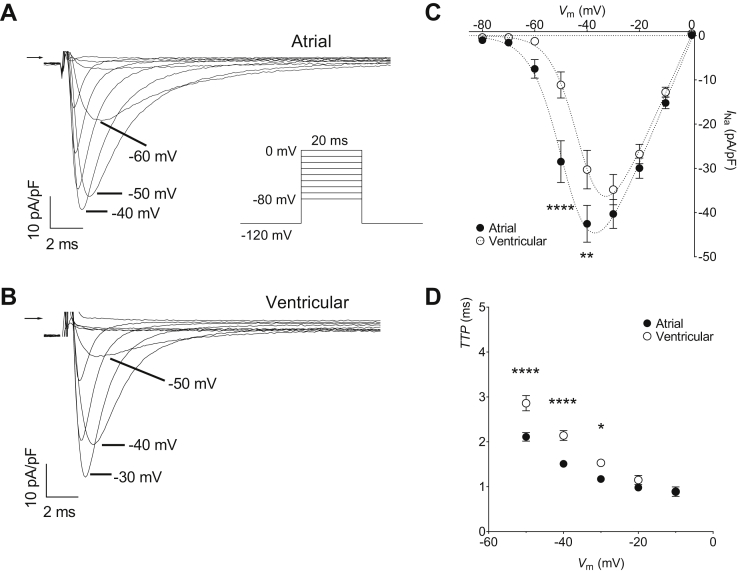
Atrial-ventricular differences in fast Na^+^ current (*I*_Na_) density–voltage relations. **A, B:** Representative current traces recorded from an atrial (A) and a ventricular myocyte (B) on depolarization to a range of voltages. Arrows indicate zero current level. Insert shows voltage pulse protocol. Residual uncompensated capacitative transients have been blanked for clarity. **C:** Mean *I*_Na_ density–voltage relations for atrial (filled circles, n = 10) and ventricular (open circles, n = 10) myocytes. Solid lines represent fits to Supplemental Equation 1. Data were significantly different by both cell type (*P* < .05) and voltage (*P* < .0001) with significant interaction (*P* < .0001; 2-way repeated measures analysis of variance). ∗∗*P* < .01; ∗∗∗∗*P* < .0001 vs ventricular; Bonferroni post hoc test. **D:** Voltage dependence of time-to-peak (TTP) *I*_Na_ for atrial (filled circles, n = 10) and ventricular (open circles, n = 10) myocytes. The membrane time constants were 0.161 ± 0.021 ms for atrial myocytes (n = 10) and 0.314 ± 0.025 ms for ventricular cells (n = 10; *P* = .00016, unpaired Student *t* test).

Depolarizing pulses activated inward currents with rapid kinetics of activation and inactivation typical of *I*_Na_ in both atrial and ventricular myocytes (Figure 1A and B). The currents of both cell types showed a U-shaped current density–voltage relation with a zero current potential close to zero mV, consistent with their Na^+^ selectivity (Figure 1C). However, *I*_Na_ from atrial myocytes activated at more negative voltages than ventricular *I*_Na_, with measurable inward currents being evident from voltages of -60 mV and positive and reaching a maximum at approximately -40 mV in atrial cells, whereas *I*_Na_ in ventricular myocytes were activated from -50 mV and reached a maximum at ∼-30 mV (Figure 1C). The whole-cell capacitance of atrial myocytes was significantly lower than that of ventricular myocytes (atrial 47.6 ± 2.5 pF, n = 10; ventricular 85.6 ± 3.7 pF, n = 10; *P* < .00001), consistent with their smaller size. The current density–voltage relations of each cell type were fitted by a modified Boltzmann relation (Supplemental Equation 1) and the more negative voltage dependence of activation of atrial *I*_Na_ was reflected in a mean half-maximal voltage of activation (*V*_*half,act*_) approximately 5.5 mV more negative than that of ventricular *I*_Na_ (*P* < .05; Supplemental Table 2). There were no significant differences between atrial and ventricular myocytes in the slope factors or maximal *I*_Na_ conductance density (Supplemental Table 2). Atrial *I*_Na_ showed shorter time-to-peak current values than ventricular currents, suggesting more rapid activation of atrial voltage-gated Na^+^ channels (Figure 1D).

**Figure 2:**
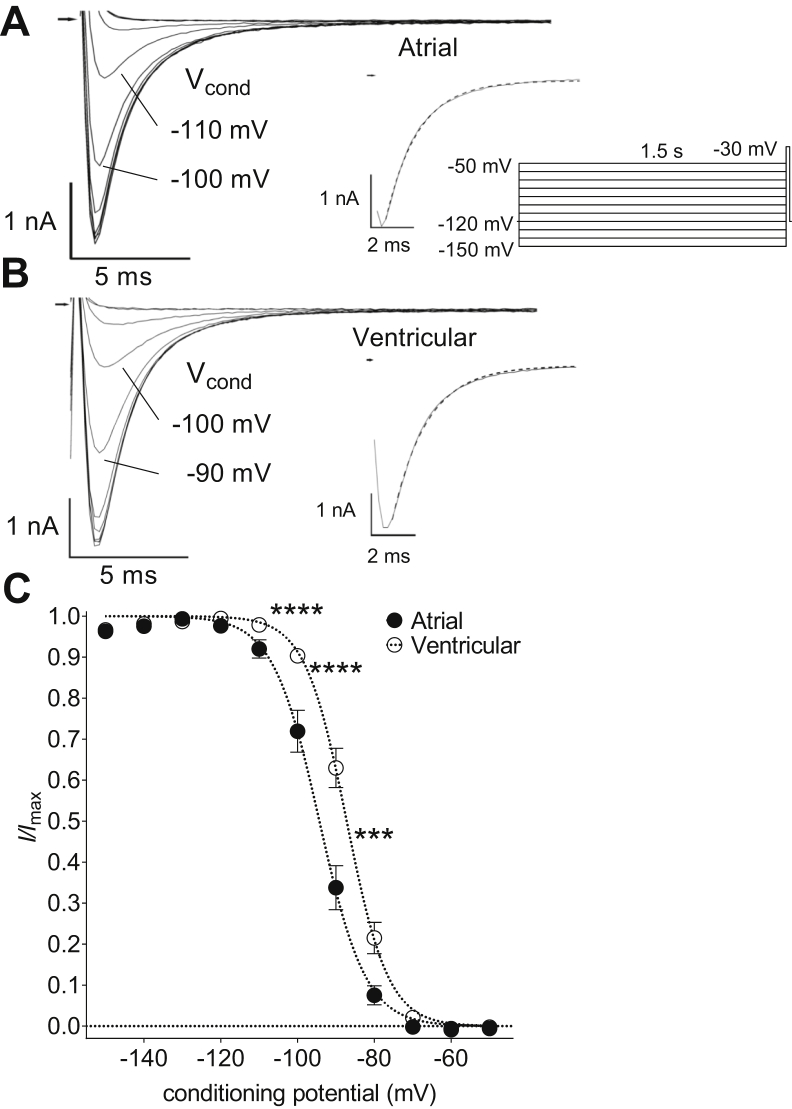
Atrial-ventricular differences in steady-state voltage-dependent inactivation of fast Na^+^ current (*I*_Na_). **A, B:** Representative current traces recorded from an atrial (A) and a ventricular myocyte (B) on depolarization to -30 mV following conditioning at a range of voltages. Conditioning potential (*V*_*cond*_) for 2 of the traces indicated. Arrows indicate zero current level. Inserts show voltage pulse protocol and current trace elicited from a *V*_*cond*_ of -130 mV on an expanded time scale. Dashed lines represent fits to Supplemental Equation 2. Supplemental Equation 2 showed a better goodness-of-fit than Supplemental Equation 3 according to the Akaike Information Criterion. Residual uncompensated capacitative transients have been blanked for clarity. **C:** Mean *I*_Na_ steady-state voltage-dependent inactivation curves for atrial (filled circles, n = 11) and ventricular (open circles, n = 12) myocytes. *I*_Na_ were normalized to the maximum inward current amplitude. Dashed lines represent fits to Supplemental Equation 4. Data were significantly different by both cell type (*P* < .001) and voltage (*P* < .0001) with significant interaction (*P* < .0001; 2-way repeated measures analysis of variance). ∗∗∗*P* < .001; ∗∗∗∗*P* < .0001 vs ventricular; Bonferroni post hoc test.

Conditioning pulses of 1.5 seconds from -150 mV to -50 mV revealed voltage-dependent inactivation of *I*_Na_ in both atrial and ventricular myocytes (Figure 2). Currents were maximal from conditioning potentials of -130 mV but showed voltage-dependent inactivation at more positive potentials. The rate of onset of *I*_Na_ inactivation was examined by fitting a single decaying exponential relation (Supplemental Equation 2) to the currents activated from a conditioning potential of -130 mV (see inserts to Figure 2A and B). There was no difference in the onset of *I*_Na_ inactivation between atrial and ventricular myocytes (τ: atrial, 1.61 ± 0.01 ms, n = 11; ventricular, 1.59 ± 0.01 ms, n = 12). Atrial *I*_Na_ inactivated at more negative voltages than the current in ventricular cells, with significant differences evident between the 2 cell types in the range from -100 mV to -80 mV (Figure 2C). The voltage dependence of inactivation was fitted by a Boltzmann relation (Supplemental Equation 4) and the mean half-maximal voltage of inactivation (*V*_*half,inact*_) of atrial cells was ∼7 mV more negative than that of ventricular myocytes (Supplemental Table 2).

**Figure 3:**
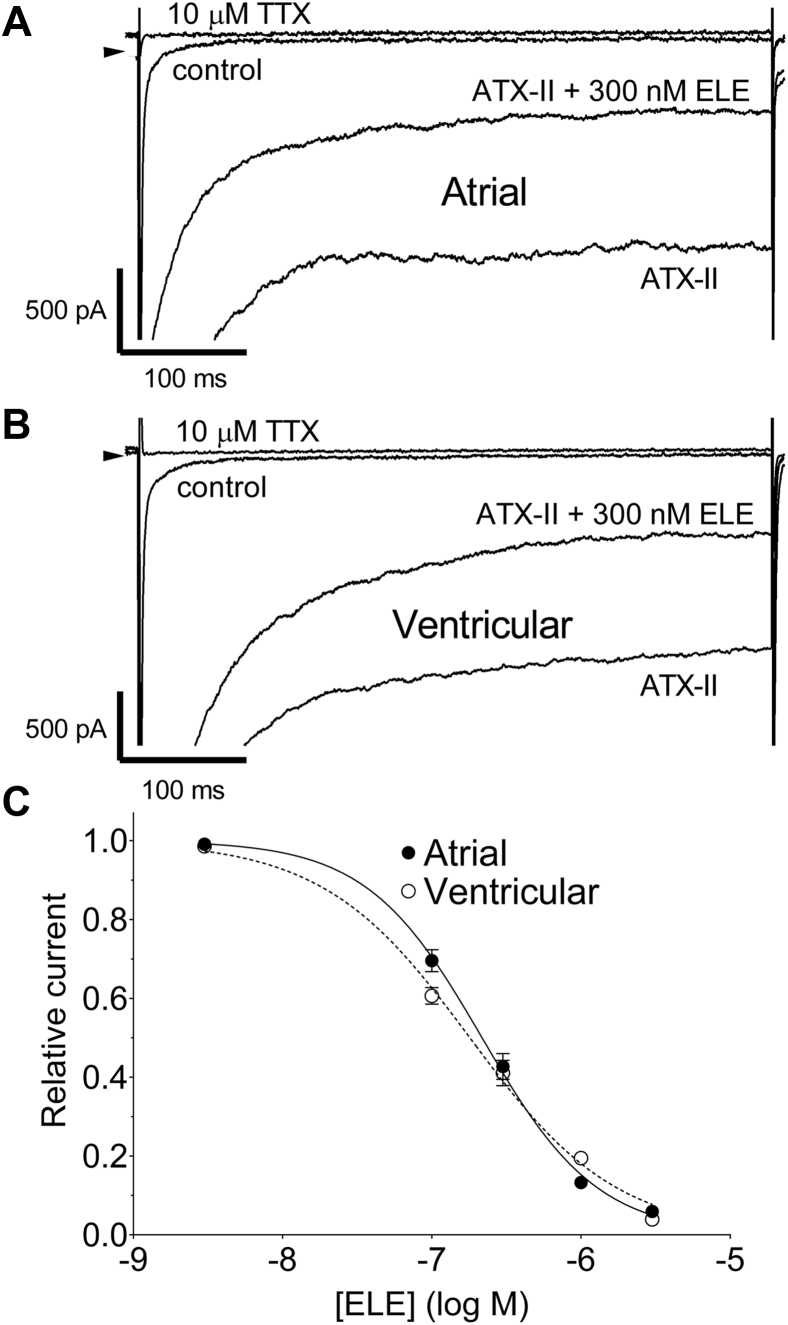
Eleclazine inhibition of the late Na^+^ current (*I*_NaL_) in atrial and ventricular myocytes. **A, B:** Representative current traces recorded from an atrial (A) and a ventricular myocyte (B) on depolarization to -20 mV in control (control), 10 μM tetrodotoxin (TTX), 3 nM sea anemone toxin (ATX-II), and eleclazine (ELE, 300 nM). **C:** Concentration dependence of *I*_NaL_ inhibition by ELE. Currents measured at 400 ms were normalized to the corresponding activated current in the presence of ATX-II alone and plotted against the corresponding concentration of ELE. Each data point represents the mean (± standard error of the mean) of data from 5 cells and each cell was exposed to a single concentration of ELE. Curves represent fits to Supplemental Equation 5.

Representative recordings of late Na^+^ currents (*I*_NaL_) activated on depolarization to -20 mV from atrial (Figure 3A) and ventricular (Figure 3B) myocytes are shown in Figure 3. Under control conditions, while there was no significant difference in the amplitude of *I*_NaL_ between the 2 cell types (atrial -20 ± 3.2 pA, n = 25; ventricular -23 ± 6.0 pA, n = 25), the atrial *I*_NaL_ density normalized to capacitance as an index of membrane surface area was almost 2-fold greater in atrial than in ventricular myocytes (atrial -0.37 ± 0.06 pA/pF, ventricular -0.19 ± 0.07 pA/pF; *P* < .05). Superfusion of the cells with 3 nM sea anemone toxin (ATX-II) caused a marked increase in *I*_NaL_ in both atrial and ventricular myocytes (*cf*. Figure 3A and B), although there was no difference between the cell types in *I*_NaL_ density in the presence of the sea anemone toxin (atrial -12.9 ± 1.2 pA/pF, n = 25; ventricular -13.8 ± 1.3 pA/pF, n = 25). The ATX-II-activated *I*_NaL_ was inhibited by ELE in a concentration-dependent manner in both atrial and ventricular myocytes (Figure 3C). There was no difference between atrial and ventricular myocytes in the concentrations of half-maximal current inhibition (*IC*_50_) by ELE (atrial 217.2 nM, ventricular 179.9 nM; Supplemental Table 3).

**Figure 4:**
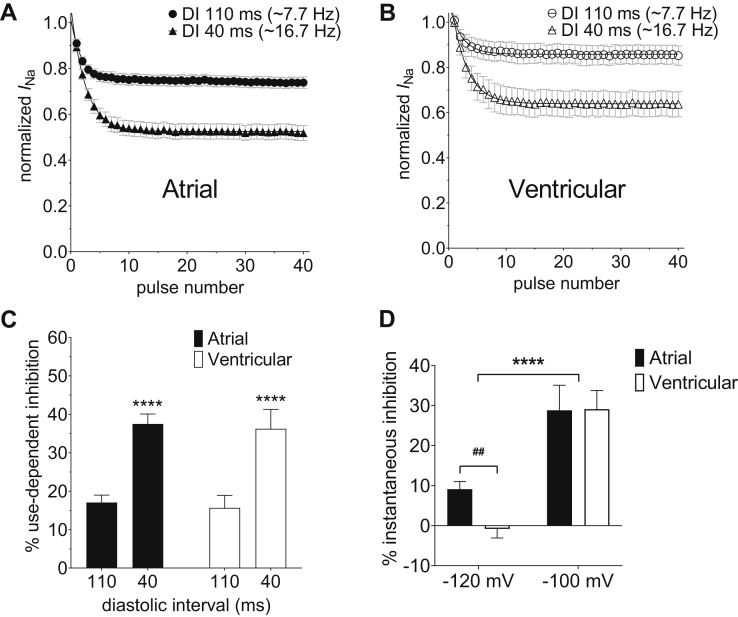
Use-dependent inhibition of fast Na^+^ current (*I*_Na_) by eleclazine (ELE, 10 μM). **A, B:** Mean normalized current amplitudes recorded by a series of 40 pulses to -30 mV at diastolic intervals (DI) of 110 (circles) and 40 ms (triangles) in atrial (A, filled symbols, n = 12) and ventricular myocytes (B, open symbols) from a holding potential of -120 mV in the presence of ELE. Currents were normalized to the currents elicited in the absence of ELE by the corresponding pulse number according to Supplemental Equation 6. Solid lines in A and B represent fits to Supplemental Equation 7. **C:** The mean percentage use-dependent inhibition (Supplemental Equation 8) at DI of 110 and 40 ms from atrial (filled columns) and ventricular (open columns) myocytes (data and sample sizes correspond to A and B). Use-dependent inhibition was significantly different by DI (*P* < .0001) but not by cell type (*P* = .7591). ∗∗∗∗*P* < .0001; Bonferroni post hoc test vs DI 110 ms in the same cell type. **D:** The effect of holding potential (HP) on mean percentage instantaneous inhibition (Supplemental Equation 9) by ELE. Data show mean inhibition on the first pulse to -30.mV at a DI of 110 ms in atrial (filled columns: HP = -120 mV, n = 12 and HP = -100 mV, n = 8) and ventricular (open columns, HP = -120 mV, n = 9 and HP = -100 mV, n = 9) myocytes. ∗∗∗∗*P* < .0001, 2-way analysis of variance. ##*P* < .01; Student unpaired *t* test.

**Figure 5:**
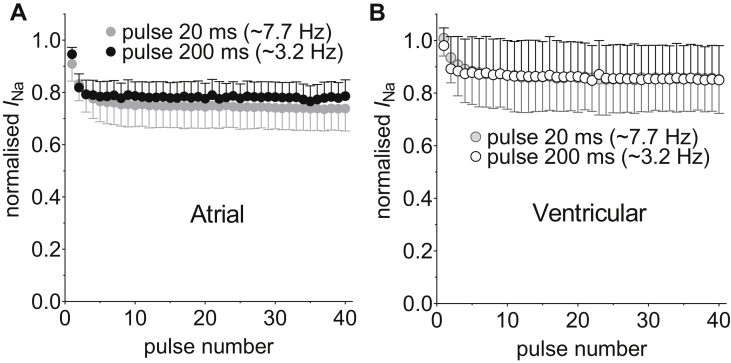
Effect of pulse duration on the use-dependent inhibition by eleclazine (ELE). Mean normalized current amplitudes recorded by a series of 40 pulses to -30 mV at diastolic intervals of 110 ms in **A:** atrial myocytes (filled circles, n = 5) and **B:** ventricular myocytes (open circles, n = 5) from a holding potential of -120 mV in the presence of 10 μM ELE. Gray-filled circles data from Figure 4 with pulse durations of 20 ms are shown for comparison (atrial n = 12; ventricular n = 9). Currents were normalized to the currents elicited in the absence of ELE by the corresponding pulse number.

The use-dependent interaction of ELE (10 μM) with voltage-gated Na^+^ channels was investigated in atrial and ventricular myocytes by examining the effects of shortening of DI on the degree of inhibition of *I*_Na_ during fixed trains of 40 consecutive pulses of 20 ms duration to -30 mV from a holding potential (HP) of -120 mV (Figure 4). The effects of DI of 110 ms and 40 ms were examined in each cell. If the drug interaction with the resting channel is weak, then ELE will tend to dissociate during the DI. Reduction of DI can thus be expected to lead to accumulation of inhibition as the time for unbinding becomes abbreviated. ELE (10 μM) inhibited *I*_Na_ in a use-dependent manner, the level of inhibition accumulating over the 40 consecutive pulses, the inhibition being greater at the shorter DI (Figure 4A and B). The rate of accumulation of inhibition by ELE was not significantly affected by DI in either atrial or ventricular myocytes and there was no difference between the 2 cell types in the rate of accumulation of inhibition (Supplemental Table 4). The use-dependent inhibition by ELE, quantified as the difference in percentage inhibition between the first and the 40th pulse, was not different between atrial and ventricular cells (Figure 4C). However, instantaneous inhibition of *I*_Na_ by ELE on the first pulse was evident in atrial but not in ventricular myocytes from the HP of -120 mV (Figure 4D). To examine the voltage dependence of the inhibition of *I*_Na_ by ELE in the 2 cell types, the fixed train protocol was run from an HP of -100 mV in separate cells (Supplemental Figure 1). There was no difference between atrial and ventricular myocytes in the use-dependent inhibition at -100 mV (Supplemental Figure 1C). However, the instantaneous inhibition by ELE was markedly increased by depolarization of the HP to -100 mV in both cell types (Figure 4D). In contrast to the data at -120 mV, there was no difference between atrial and ventricular myocytes in the instantaneous inhibition of *I*_Na_ at -100 mV. Consequently, although the total inhibition, as calculated according to Supplemental Equation 10, by ELE evident on the final pulse at either DI was greater in atrial than in ventricular myocytes at -120 mV, there was no difference in total inhibition between the 2 cell types at -100 mV (Supplemental Figure 2). To examine whether ELE interacts strongly with inactivated states of the Na^+^ channel, we followed the methodology of Zygmunt and colleagues^16^ and examined the effect on use-dependent inhibition of prolonging the depolarizing pulse to 200 ms at a constant DI of 110 ms (Figure 5). Prolongation of the depolarizing pulse from 20 to 200 ms can be expected to have increased the proportion of channels in the inactivated state at the end of the pulse.^16^ The total inhibition was unaffected by pulse duration in either atrial or ventricular myocytes (2-way analysis of variance [ANOVA]), consistent with preferential association of ELE with activated states of the channel.

**Figure 6:**
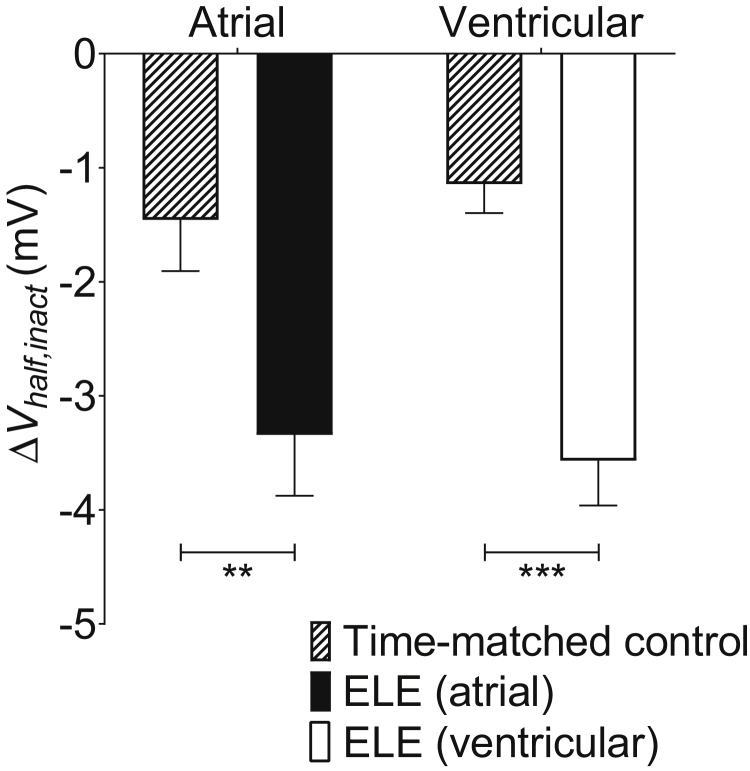
Effect of eleclazine (ELE) on half-maximal voltage of steady-state inactivation. Data shown are the mean changes in half-maximal voltage of inactivation (*V*_half,inact_) caused by 10 μM ELE for atrial (filled column, n = 9) and ventricular (open column, n = 9) myocytes. Hatched columns show corresponding time-matched controls in the absence of ELE for 8 atrial and 9 ventricular myocytes. ∗∗*P* < .01; ∗∗∗*P* < .001; 2-way analysis of variance with Bonferroni post hoc test.

The effect of ELE on steady-state inactivation was examined as the ELE-induced shift in the half-maximal voltage of inactivation (Δ*V*_*half,inact*_) in atrial and ventricular myocytes (Figure 6). In both cell types, treatment with ELE was associated with a negative shift in *V*_*half,inact*_ of ∼3.5 mV (Figure 6). Time-matched control experiments showed a time-dependent shift in *V*_*half,inact*_ in atrial and ventricular myocytes, with no significant difference between the cell types in the magnitude of the shift (Figure 6). Although the ELE-induced shift in *V*_*half,inact*_ was significantly greater than the time-matched control in both atrial and ventricular myocytes, there was no significant difference between cell types in the drug-induced shift in *V*_*half,inact*_.

**Figure 7:**
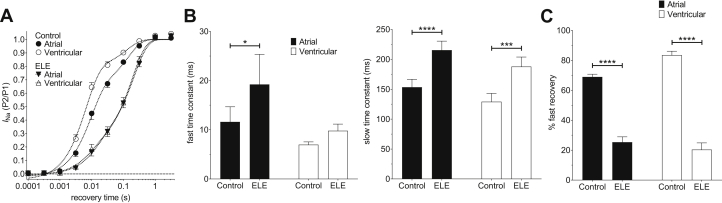
Effect of eleclazine (ELE) on fast Na^+^ current (*I*_Na_) recovery from inactivation. **A:** Recovery of *I*_Na_ from inactivation in atrial (filled symbols, n = 7) and ventricular (open symbols, n = 7) myocytes. Dashed lines represent fits to Supplemental Equation 11. The holding potential during recovery was -120 mV. **B:** Fitted fast (left-hand panel) and slow (right-hand panel) time constants in control and in the presence of 10 μM ELE for atrial (filled columns) and ventricular (open columns) myocytes. ∗*P* < .05; ∗∗∗*P* < .001; ∗∗∗∗, *P* < .0001; 2-way repeated measures analysis of variance (ANOVA) with Bonferroni post hoc test vs control. **C:** Mean amplitude of fast component in control and in the presence of 10 μM ELE for atrial (filled columns) and ventricular (open columns) myocytes. ∗∗∗∗*P* < .0001; 2-way repeated measures ANOVA with Bonferroni post hoc test vs control.

**Figure 8:**
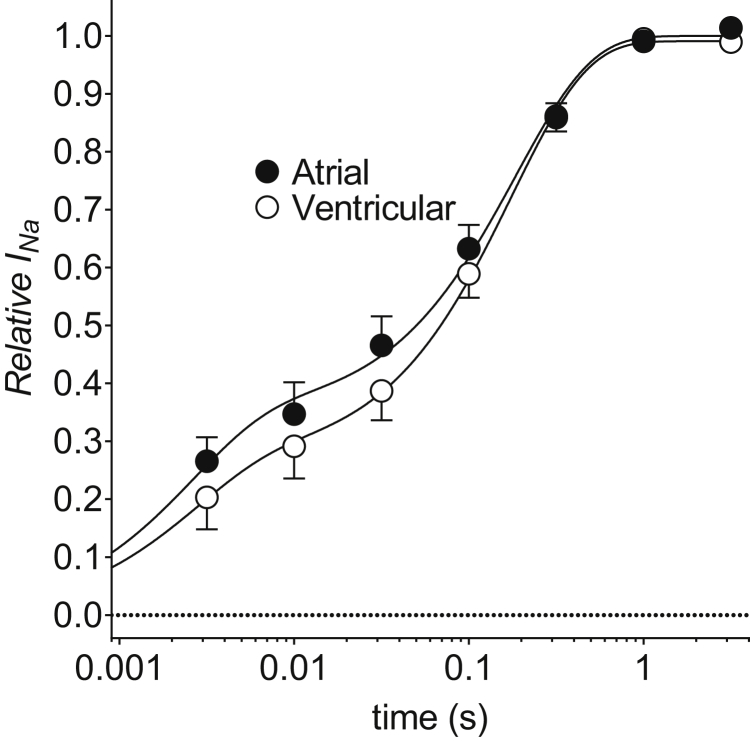
The time course of recovery from eleclazine binding. Data were calculated from the data shown in Figure 7A using Supplemental Equation 12. Filled circles, atrial myocytes (n = 7); open circles, ventricular myocytes (n = 7). Solid lines represent fits to Supplemental Equation 11. Fits were constrained to go to 0 at time = 0.0001 second. Fitted parameters are shown in Supplemental Table 6.

In both atrial and ventricular myocytes, *I*_Na_ recovered from inactivation with a biexponential time course, with a fast time constant (*τ*_*f*_) of 5–30 ms and a slow time constant (*τ*_*s*_) of 100–220 ms (Figure 7A). Under control conditions, the recovery from inactivation was slower in atrial than in ventricular myocytes (*P* < .0001, 3-way ANOVA; Supplemental Table 5). Although the mean fast and slow time constants were larger (ie, slower) in atrial than in ventricular cells, this did not achieve the level of statistical confidence (Figure 7B). On the other hand, although the fast component of recovery predominated in both cell types, the contribution of the fast component was less in atrial than in ventricular myocytes (Figure 7C). The recovery from inactivation was slowed by ELE in both cell types. The slowing of the mean fast time constant was greater in atrial (∼65% increase) than in ventricular (∼40% increase) myocytes, whereas there was little difference between atrial and ventricular myocytes in the effect of ELE on the slow time constant (40%–45% increase in both cell types) (Figure 7B). ELE decreased the contribution of the fast component to recovery to 20%–25% in both cell types, so that the mean time course of recovery from inactivation of *I*_Na_ was indistinguishable in the 2 cell types in the presence of the drug (Figure 7A and C). There was no significant difference between atrial and ventricular myocytes in the rates of recovery from the drug-bound state (Figure 8). In both atrial and ventricular myocytes, the dissociation of ELE occurred in 2 phases; a fast phase with time constant (τ_f_) ∼2.5 ms and a slow phase with τ_s_ ∼180 ms (Supplemental Table 6). The time constants of dissociation did not differ between atrial and ventricular myocytes.

ELE was found to prolong the left atrial ERP in the presence of the muscarinic agonist carbachol (CCh) in Langendorff-perfused rat hearts (Supplemental Figure 3A). Perfusion with CCh (0.5–0.75 μM) shortened ERP by ∼56% (*P* < .01). Subsequent perfusion with ELE (1 μM) in the continued presence of CCh resulted in recovery of ERP to ∼60% of the control value (*P* < .05). On the other hand, neither CCh nor ELE had significant effect on CV (Supplemental Figure 3B). Taken together, these data are consistent with inhibition of atrial *I*_Na_ by ELE.

## Discussion

### Atrial-ventricular differences in *I*_Na_

Clear atrial-ventricular differences were found in the biophysical properties of rat cardiac voltage-gated Na^+^ currents: atrial *I*_Na_ was activated at command potentials ∼5.5 mV more negative and inactivated at conditioning potentials ∼7 mV more negative than ventricular *I*_Na_, the activation time-to-peak *I*_Na_ following depolarization to voltages of -50 to -30 mV was also shorter in atrial than in ventricular myocytes, and the recovery of *I*_Na_ from inactivation was significantly slower in atrial than in ventricular myocytes. In these respects, the differences in *I*_Na_ between rat atrial and ventricular cells in the present study were similar to those reported previously in cardiac myocytes from various species.^7^^,^^9^, ^10^, ^11^, ^12^ On the other hand, in contrast to previous reports from the dog, guinea pig, and rabbit demonstrating greater *I*_Na_ density in atrial compared to ventricular myocytes, there was no difference in *I*_Na_ density between the 2 cell types in the present study.^7^^,^^9^^,^^12^ Under control conditions, the density of *I*_NaL_ was greater in atrial than in ventricular myocytes. Atrial-ventricular differences in the inhibition of *I*_Na_ by ELE were also evident: (1) in addition to marked use-dependent inhibition that was not significantly different between the 2 cell types, ELE caused an instantaneous inhibition of *I*_Na_ in atrial and ventricular myocytes that was dependent on membrane potential, and consequently arose at more negative membrane potentials in atrial myocytes than in ventricular myocytes; and (2) there was an atrial-selective slowing of the fast time constant of recovery from inactivation. To the best of our knowledge, this represents not only the first report of atrial-ventricular differences in the effects of ELE on *I*_Na_ from any species, but the first report of atrial-ventricular differences in inhibition of peak *I*_Na_ by any *I*_NaL_ inhibitor with rapid kinetics other than ranolazine. Consistent with inhibition of peak *I*_Na_, ELE partially reversed the shortening in atrial ERP induced by the muscarinic agonist CCh, indicating an atrial antiarrhythmic action of the drug. On the other hand, it is unclear whether the effect of ELE was atrial-selective.

### Mechanism of eleclazine inhibition

ELE has been shown to block cardiac Na^+^ channels with unusually rapid kinetics, interacting with the local anesthetic binding site and showing marked selectivity for *I*_NaL_ relative to fast *I*_Na_.^28^ The present study demonstrates that there was no difference between atrial and ventricular myocytes in the concentration-dependent inhibition of ATX-II-activated *I*_NaL_ by ELE, indicating similar affinity of ELE for atrial and ventricular Na^+^ channels. Our data are consistent with the inhibition of *I*_NaL_ by ELE via open channel block, as suggested in previous reports.^26^, ^27^, ^28^, ^29^ The inhibition of both *I*_Na_ and *I*_NaL_ by ELE has been shown to be voltage-dependent so that the IC_50_s for inhibition of the ATX-II-activated *I*_NaL_ in atrial and ventricular myocytes in the present study (217 nM and 180 nM, respectively) were comparable with the value reported previously in rabbit ventricular myocytes at a corresponding HP of -80 mV (260 nM).^27^ Consistent with previous reports on recordings of fast *I*_Na_ from rabbit ventricular myocytes and recombinant human Na_v_1.5 channels,^27^^,^^28^ 10 μM ELE produced significant use-dependent inhibition, with little difference between atrial and ventricular myocytes. The rates of accumulation of *I*_Na_ inhibition evident during the repeated pulse protocol in the present study (Figure 4) were very much faster than those reported previously for ranolazine in canine and rabbit cardiac myocytes using similar pulse widths, DI, and numbers of pulses, consistent with the unusually rapid kinetics of ELE.^12^^,^^16^ Though therapeutically effective doses of ELE produce plasma concentrations of ∼0.5 μM, ELE is highly lipophilic (logP ∼4.35) and accumulates at higher concentrations in cardiac tissue.^33^ Thus, it is conceivable that treatment with ELE would affect fast *I*_Na_, particularly at higher rates during tachyarrhythmias.

The accentuation of use-dependent inhibition of fast *I*_Na_ at shorter diastolic intervals in the present study reflected incomplete recovery from block during the diastolic interval when the channels tend to return to the resting state. The data are consistent with preferential interaction of ELE with activated states of both atrial and ventricular Na^+^ channels. Increasing the duration of the depolarizing pulse had no effect on the total inhibition by ELE, indicating only weak interaction of the drug with the inactivated state of the channel in either cell type. In the present study, the dissociation of the drug from Na^+^ channels was exceptionally rapid: dissociation of the drug followed a biexponential time course with time constants of ∼2.5 ms and ∼180 ms, with no appreciable differences between atrial and ventricular myocytes. The value of the slow time constant of dissociation was similar to that of the slow component of recovery from inactivation (τ_s_ ∼130 ms in both cell types), consistent with trapping of the drug by the inactivation gate impeding dissociation of ELE from the channel. The degree of instantaneous inhibition by ELE in atrial and ventricular myocytes at an HP of -100 mV, at which there was very little *I*_Na_ activation in either cell type, was striking. From an HP of -120 mV, the instantaneous inhibition of *I*_Na_ was markedly reduced in both cell types, becoming negligible in ventricular myocytes. Although a hydrophobic pathway for access of neutral drugs to the local anesthetic binding site of Na^+^ channels has been suggested, the relatively large size of ELE (molecular weight ∼415.4 g.mol^-1^), comparable to ranolazine, argues against significant access via that route.^15^^,^^34^ On the other hand, overlap in the voltage dependence of steady-state activation and inactivation will result in a small, but nevertheless significant, window current at negative voltages (Supplemental Figure 4). The instantaneous inhibition in atrial myocytes at -120 mV therefore likely results from the small proportion of channels activated at the HP. Thus, the difference between atrial and ventricular myocytes in instantaneous inhibition likely reflects differences in the voltage dependence of activation of the window current in the 2 cell types.

Given the use-dependent action of ELE, its preference for the activated state, and rapid dissociation from the noninactivated sodium channel, the less negative resting membrane potential, differences in action potential morphology, and consequent shorter diastolic interval of atrial relative to ventricular myocytes may contribute to an atrial-selective action of ELE.^7^^,^^16^

## Conclusions

Although there was no evidence of any difference between atrial and ventricular *I*_Na_ (or *I*_NaL_) in the kinetics of drug binding/dissociation, the more negative voltage dependence of *I*_Na_ activation and inactivation underlies an atrial-selective instantaneous inhibition of *I*_Na_ by ELE. The present study provides further evidence to support the contention that *I*_NaL_ inhibitors have differential effects on atrial and ventricular *I*_Na_. However, additional future investigation is now required to establish whether the actions of ELE on atrial *I*_Na_ form the basis for an atrial-selective antiarrhythmic action. Chamber-specific human induced pluripotent stem cell–derived cardiomyocytes (hiPSC-CM) represent an attractive potential human model for validation of the atrial-selective action of antiarrhythmic drugs. However, though differentiation protocols are available to drive hiPSCs towards an atrial- or ventricular-like phenotype, iPSC-CM express both fetal (exon 6A) and adult (exon 6) isoforms of SCN5A, leaving the relevance of the data from studies using these models open to question.^35^ On the other hand, studies in a pertinent model of AF would provide important evidence regarding the potential for an atrial-selective antiarrhythmic action of ELE.
