# Supplementary material for: Inhibition of voltage-gated Na+ currents by eleclazine in rat atrial and ventricular myocytes
Source: Heart Rhythm O2. 2020 May 25;1(3):206–14. doi: 10.1016/j.hroo.2020.05.006 (PMC7442036; doi:10.1016/j.hroo.2020.05.006)
Supplement: Supplementary Material [file mmc1.docx]

**Supplemental Information Appendix**

**Methods**

***Rat cardiac myocyte isolation***

Male Wistar rats (230 – 280 g) were sacrificed in accordance with UK Home Office legislation using methods approved by the *Animal Welfare and Ethics Review Board* of the University of Bristol. Left ventricular and left atrial myocytes were isolated from 59 rats by enzymatic and mechanical dispersion as described previously^1,2^. Cells were stored in Kraftbrühe (KB) solution at 4 °C until use^3^.

Rats were chosen as a model species as they have the benefit of having little contribution of the rapid component of the delayed rectifier (*I*_Kr_) to atrial and ventricular repolarization^4^. Thus, the rat represents a suitable species in which to examine the atrial antiarrhythmic action of *I*_Na_ inhibition by the selective *I*_NaL_ inhibitor eleclazine in the absence of *I*_Kr_ inhibition^5^.

***Whole-cell recording of voltage-gated Na^+^ currents***

Cells were placed in an experimental chamber mounted on the stage of an inverted microscope (Diaphot 200, Nikon UK Ltd, UK) and superfused with a Tyrode’s solution containing (in mM) NaCl 140, KCl 4, CaCl_2_ 1.2, MgCl_2_ 1, HEPES 5 and D-glucose 10 at room temperature (pH 7.35 with NaOH). Whole-cell voltage-clamp recordings were made using an EPC-9 amplifier (HEKA GmbH, Germany). Patch-pipettes (A-M Systems, USA) were pulled to a final resistance of 1-2 MΩ (P-97 Flaming/Brown Puller, Sutter Instruments, USA). Protocols were generated and data recorded on-line with Pulse software (v8.11, HEKA GmbH, Germany). Currents were subject to two low-pass 4-pole Bessel filters (*f*_c1_=10 kHz, *f*_c2_=2.9 kHz). Sampling frequencies were typically 10 – 100 kHz unless otherwise specified. Internal (pipette) and external solutions for recording voltage-gated fast Na^+^ currents (*I*_Na_) and the late component of the Na^+^ current (*I*_NaL_) are shown in Supplemental Table 1. The junction potential was compensated electronically on immersion of the pipette tip in the bath solution and no further compensation was applied. Mean whole-cell capacitances were: atrial myocytes, 50±1.6 pF (*n*=71) and ventricular myocytes, 128±4.4 pF (*n*=81). Mean series resistances were: atrial, 3.7±0.1 MΩ and ventricular, 4.1±0.1 MΩ. Series resistance compensation was applied and was typically 50 – 60 %. No further correction was made for voltage-drop error, which was estimated to be between -1 to -6 mV at the maximal inward currents in both cell types.

***Voltage command protocols and data analysis***

**Data presented in Figure 1**. *I*_Na_-voltage relations were obtained from atrial and ventricular myocytes by step depolarizations (20 ms duration, 1/5 s) to voltages of -80 mV to 0 mV from a holding potential of -120 mV. *I*_Na_ was measured as the difference between the peak inward current and the current at the end of the pulse. Currents were normalized to whole-cell capacitance as an index of cell surface area and expressed in pA/pF. Mean *I*_Na_ densities were plotted against the corresponding command voltage (*V_m_*) and fitted with a modified Boltzmann equation^6^:

*Supplemental Equation 1*. $I_{Na}\left( V_{m} \right)=\frac{G_{max}.\left( V_{m}-V_{rev} \right)}{\left( 1+exp\left( \frac{\left( V_{m}-V_{half,act} \right)}{k} \right) \right)}$ ,

where *G_max_* represents the maximal Na^+^ conductance, *V_rev_* is the effective reversal potential, *V_half,act_* is the voltage of half-maximal current activation and *k* is a slope factor.

**Data presented in Figure 2**. Steady-state voltage-dependent inactivation was examined by application of 1.5 s conditioning commands from -150 mV to -50 mV prior to activation of *I*_Na_ by a 20 ms depolarizing pulse to -30 mV (1/10 s). Currents were sampled at 5 kHz. The time course of *I*_Na_ inactivation following the peak inward current was fitted with both single and double decaying exponential equations, as follows:

*Supplemental Equation 2*. $I_{Na}\left( t \right)=A.\exp\left( -\frac{t}{\tau} \right)+c$ ,

where *τ* is the single time constant of inactivation and *c* is a non-inactivating leak current.

*Supplemental Equation 3*. $I_{Na}\left( t \right)=A_{f}.\exp\left( -\frac{t}{\tau_{f}} \right)+A_{s}.\exp\left( -\frac{t}{\tau_{s}} \right)+c,$

where *τ_f_* and *τ_s_* are, respectively, the fast and slow time constants of inactivation, and *A_f_* and *A_s_* are the amplitudes of the fast and slow components of inactivation. The goodness-of-fit was assessed using the Akaike Information Criterion (AIC)^7^. The fit giving the lowest AIC was selected. In all cases, this corresponded to a fit to *Supplemental Equation 2*.

*I*_Na_, measured as the difference between the peak inward current and the current at the end of the pulse, was normalized to the current from a conditioning potential of -130 mV (*I*_max_) and plotted against the corresponding conditioning potential. Steady-state inactivation curves were fitted with a Boltzmann relation, as follows:

*Supplemental Equation 4*. $\frac{I}{I_{max}}(V_{m})= \frac{1}{(1+exp\left( \frac{\left( V_{half,inact}-V_{m} \right)}{k} \right))}$ ‘

where *V_half,inact_* is the voltage of half-maximal inactivation and *k* is a slope factor.

**Data presented in Figure 3**. For the recording of *I*_NaL_, whole-cell currents were activated by a 500 ms depolarization to -20 mV from a holding potential of -80 mV. *I*_NaL_ was measured as the current sensitive to 10 μM tetrodotoxin (TTX) at 400 ms following depolarization. Under control conditions in both atrial and ventricular myocytes, *I*_NaL_ were very small (Figure 3). Cells were superfused with 3 nM of the sea anemone toxin, ATX-II, slowing Na^+^ current inactivation and thereby increasing *I*_NaL_ for the assessment of the concentration dependence of current inhibition, as others have done previously^5,8-11^. Cells were subsequently superfused with eleclazine (ELE) at concentrations from 3 nM to 3 μM in the continued presence of ATX-II. Each cell was exposed to a single concentration of ELE. The binding sites for ATX-II and ELE are not thought to overlap^10,12^ and it is unlikely that allosteric interaction between ATX-II and ELE contributed to the inhibition of *I*_NaL_. The concentration-dependence of *I*_NaL_ inhibition was examined by plotting the ratio of current in the presence of ELE (*I*_ELE_) to the corresponding control current (*I*_control_) in the presence of ATX-II against the corresponding concentration of ELE (Fig. 3C). The data were fitted with the following equation:

*Supplemental Equation 5*. $\frac{I_{ELE}}{I_{control}}=\frac{1}{1+{10}^{(\left( LogIC50-\left[ ELE \right] \right)*n_{H})}}$

where *LogIC50* is the log_10_ of the half-maximal inhibitory concentration (*IC*_50_), *[ELE]* is the concentration of eleclazine and *n*_H_ is the Hill coefficient.

**Data presented in Figure 4, Figure 5, Supplemental Figure 1 and Supplemental Figure 2**. The use-dependent block of fast *I*_Na_ by ELE was examined using a protocol developed from previous reports^13,14^ consisting of a consecutive series of 40 depolarizing pulses to -30 mV (either 20 ms or 200 ms, as indicated in the figure) applied at diastolic intervals (DI) of 110 and 40 ms. Currents were obtained by 40 consecutive pulses in control solution and then superfused with 10 μM ELE for ~30 s without stimulation before repeating the protocol in the continued presence of ELE. The concentration of ELE used was selected according to previous studies reporting use-dependent block of fast *I*_Na_ by ELE^10,11^. Currents in the presence of ELE were normalized to the current obtained by the corresponding pulse number in the absence of ELE according to the following equation:

*Supplemental Equation* 6. $ratio (n)= \frac{I_{Na}(n, drug)}{I_{Na}(n, control)}$,

where *ratio (n)* represents the normalized current at the *n*^th^ pulse, *I*_Na_ (*n,drug*) was the current at the *n*^th^ pulse in the presence of ELE and *I*_Na_ (*n,control*) was the current at the *n*^th^ pulse in control solution.

The *ratio* (*n*) was plotted against the pulse number and the time course of use-dependent inhibition was fitted by the following equation:

*Supplemental Equation 7*. $ratio \left( n \right)=b_{ss}+\left( Y_{0}-b_{ss} \right)e^{\left( -K\times n \right)}$,

where *n* is the number of pulses, *b_ss_* is the steady-state inhibition, *Y_0_* is the intercept at the y-axis and *K* is the uptake rate (in pulses^-1^), as described previously^13^.

The use-dependent inhibition (*UDI*) by ELE was quantified using the following equation:

*Supplemental Equation 8.*

$UDI \left( \% \right)=\left( ratio \left( 1^{st} pulse \right)-ratio \left( {40}^{th} pulse \right) \right)\times100$.

The instantaneous inhibition by ELE was quantified as follows:

*Supplemental Equation 9*.

$instantaneous inhibition \left( \% \right)=\left( 1-ratio \left( 1^{st} pulse \right) \right)\times100$.

The percentage total inhibition was calculated as follows:

*Supplemental Equation 10.*

$total inhibition \left( \% \right)=instantaneous inhibition \left( \% \right)+UDI (\%)$

**Data presented in Figure 7**. To examine the recovery of *I*_Na_ from inactivation, currents were activated and inactivated by a 1 s pulse to -30 mV from a holding potential -120 mV followed by a second pulse to -30 mV (10 ms) with a delay that varied from 0.1 to 3162 ms in half-log intervals. Currents were normalized to the current activated by the first pulse (*I_control_*) and plotted against the interval time. Data were fitted with the following equation.

*Supplemental Equation 11*.

$\frac{I}{I_{control}}\left( t \right)=1-(A_{f}.\exp\left( \frac{-t}{\tau_{f}} \right)+{(1- A}_{f}).\exp\left( \frac{-t}{\tau_{s}} \right))$ ,

where *τ_f_* and *τ_s_* are, respectively, the fast and slow time constants of recovery from inactivation and *A_f_* and *A_s_* are the corresponding amplitudes of the fast and slow components of recovery.

**Data presented in Figure 8**. Data obtained using the protocol described in the previous paragraph were used to examine the time-dependence of recovery from the drug-bound state. The recovery of drug-bound channel currents obtained at time intervals from 3.162 to 3162 ms were isolated from the recovery of non-drug-bound channel currents using the following equation^10^.

*Supplemental Equation 12*.

$\frac{I}{I_{control}}(t)=\frac{\frac{I_{drug\_rec}}{I_{drug\_max}}\left( t \right)}{\frac{I_{control\_rec}}{I_{control\_max}}} (t)$,

where *I_control_rec_*/*I_control_max_*(*t*) represents the proportion of the current recovered at each time interval (*t*) in the absence of drug and *I_drug_rec_*/*I_drug_max_*(*t*) represents the corresponding data in the presence of the drug^10^.

***Electrophysiological recordings from whole hearts.***

Hearts were excised under general anesthesia (80-100 mg/kg sodium pentobarbital, IP), mounted on a whole heart perfusion apparatus and perfused retrogradely at a rate of 8 ml.min^-1^.g^-1^ heart weight via the aorta with a Krebs’ Henseleit (KH) solution (in mM; 118.5 NaCl, 25.0 NaHCO_3_, 3.0 KCl, 1.2 MgSO_4_.7H_2_O, 1.2 KH_2_PO_4_, 2.5 CaCl_2_, 11.1 D-glucose at 37^o^C) gassed with 95% O_2_/5% CO_2_, as described previously^15-17^. Left atrial unipolar electrograms were recorded using a 4×5 array of recording electrodes^16^. The electrode array was pressed against the epicardial surface of the anterior aspect of the left atrial free wall. The interelectrode distance was 334 µm; therefore, the recording area was 1.002 mm × 1.336 mm. All electrograms were individually amplified and were acquired to a PC hard disk via a Power1401 interface using Spike 2 software (Cambridge Electronic Design, UK). Bipolar stimulation was applied via electrodes adjacent to the recording array and stimulus protocols were generated using an AMPI Master-8 programmable stimulator with Iso-Flex stimulus isolators (Intracel Ltd., UK). Atrial effective refractory period (ERP) was measured using an S_1_-S_2_ stimulus protocol^15,16^. The activation time at each electrode was taken as the time at the point of maximum negative deflection and was measured relative to the earliest fiducial point of activation^16-18^. The maximal vectorial conduction velocity (CV) across the array was calculated from the electrogram most distal, and with the shortest activation time, relative to the earliest fiducial point^16-19^. Hearts (*n*=5) were perfused with control solution and, after an equilibration period, ERP and CV measured at a cycle length (CL) of 100 ms. Each heart was then perfused with carbachol (CCh) (0.5 – 0.75 µM) and, once a steady-state had been achieved, the measurements repeated. The hearts were then perfused with 1 µM eleclazine (ELE) in the presence of CCh and the measurements repeated once again.

***Eleclazine***

Eleclazine (ELE) was supplied as a gift by Dr James T. Milnes (Xention Ltd, Cambridge, UK) and made as a stock solution in DMSO, stored at -20 °C and diluted to the final concentration in low Na^+^ external recording solution on the day of experiment.

***Statistics***

Data are presented as the mean ± standard error of the mean. Unless specified, comparisons between atrial and ventricular myocytes were made by Student’s unpaired t-test or two-way ANOVA with Bonferroni *post hoc* tests using Prism v7.04 (GraphPad Software Inc, CA, USA). Current density-voltage relations and the voltage-dependence of steady-state inactivation were compared by two-way repeated measures (RM) ANOVA and Bonferroni *post hoc* tests, with voltage as the repeated measure. The use-dependent and instantaneous block by ELE in the two cell types were analyzed by two-way ANOVA with Bonferroni *post hoc* tests. The time course of recovery from inactivation was analyzed by three-way ANOVA, with the factors being time, cell type and drug-treatment. The effect of CCh and ELE on AERP and CV in Langendorff-perfused hearts were analyzed by repeated measures one-way ANOVA with Tukey *post hoc* test. P<0.05 was used as the limit of statistical confidence. Curve fitting was performed by non-linear least squares using Igor Pro v6 (Wavemetrics Inc, OR, USA).

**Supplemental Figures**


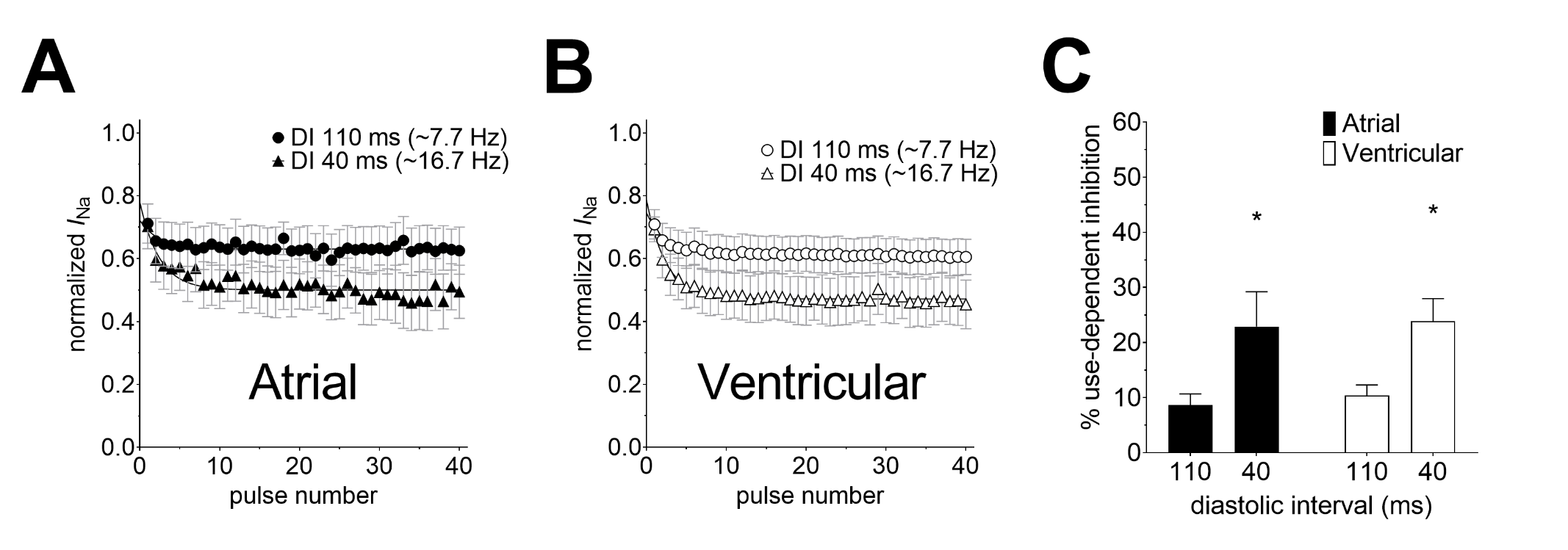


**Supplemental Figure 1**. Use-dependent block of *I*_Na_ by eleclazine (ELE, 10 μM) from a holding potential of -100 mV. **A:** Mean normalized current amplitudes recorded by a series of 40 pulses to -30 mV at diastolic intervals (DI) of 110 (circles) and 40 ms (triangles) in atrial myocytes (filled symbols, *n*=8) in the presence of ELE. Currents were normalized to the currents elicited in the absence of ELE by the corresponding pulse number. **B:** Mean normalized current amplitudes recorded using the same protocol as used in **A** from ventricular myocytes (open symbols) in the presence of ELE (*n*=9). Solid lines in **A** & **B** represent fits to *Supplemental Equation 6*. **C:** The mean percentage use-dependent block at DI of 110 and 40 ms from atrial (filled columns) and ventricular (open columns) myocytes (sample sizes correspond to **A** & **B**). Use-dependent block was significantly different by DI (P<0.0001) but not by cell type (P=0.7591). *, P<0.05; Bonferroni *post hoc* test vs DI 110 ms in the same cell type.


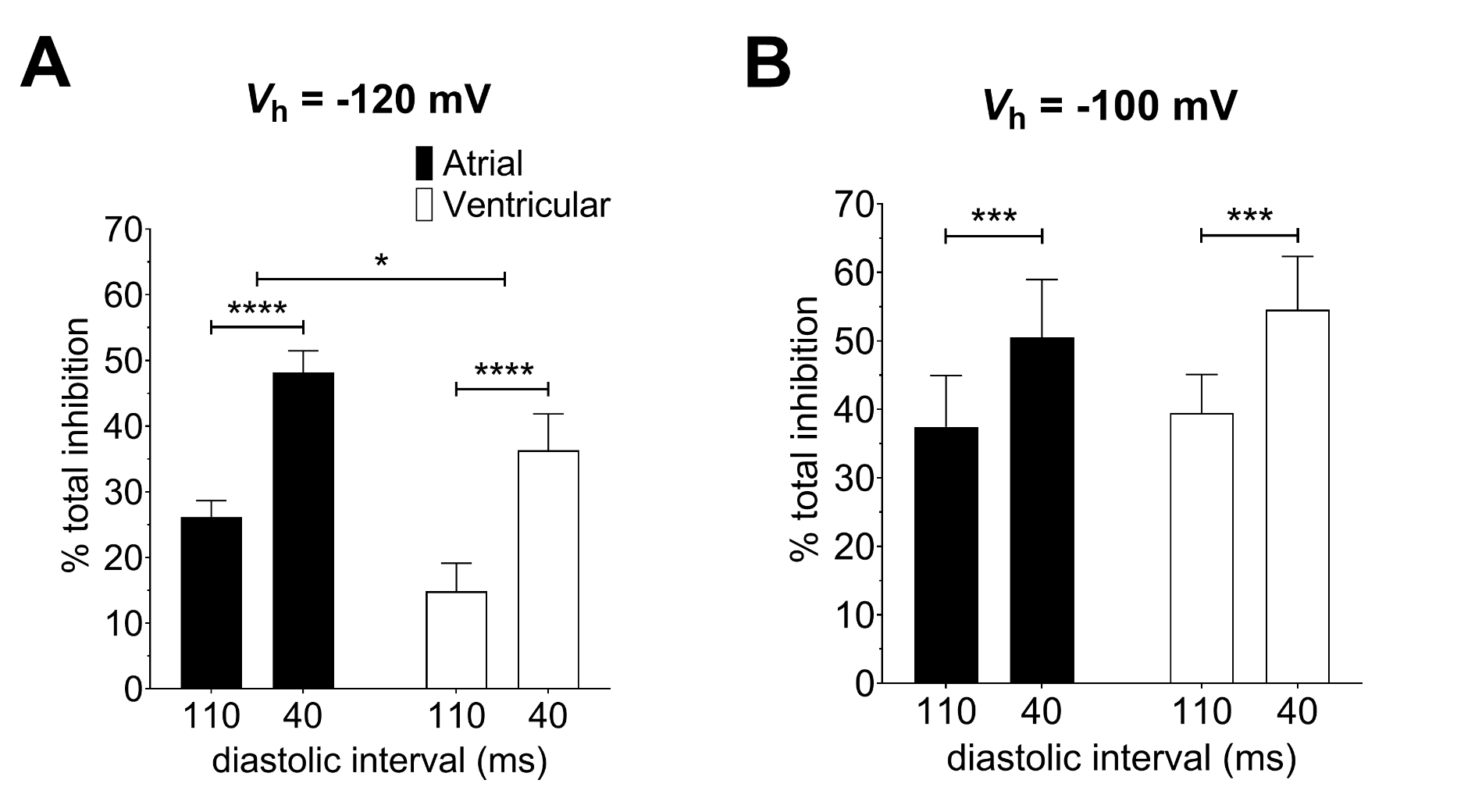


**Supplemental Figure 2**. The effect of holding potential on the percentage total inhibition by 10 µM ELE. Percentage total inhibition was calculated according to *Supplemental Equation 10*. **A** Mean percentage total inhibition by ELE of *I*_Na_ in atrial (black columns, *n*=12) and ventricular (white columns, *n*=9). Data correspond to those shown in Figure 4, panels A & B. Data were significantly different by diastolic interval (****, P<0.0001) and cell type (*, P<0.05, two-way ANOVA with repeated measures for diastolic interval). **B** Mean percentage total inhibition by ELE of *I*_Na_ in atrial (black columns, *n*=8) and ventricular (white columns, *n*=9). Data correspond to those shown in Supplemental Figure 1. Data were significantly different by diastolic interval (***, P<0.001) but not by cell type (two-way ANOVA with repeated measures for diastolic interval).


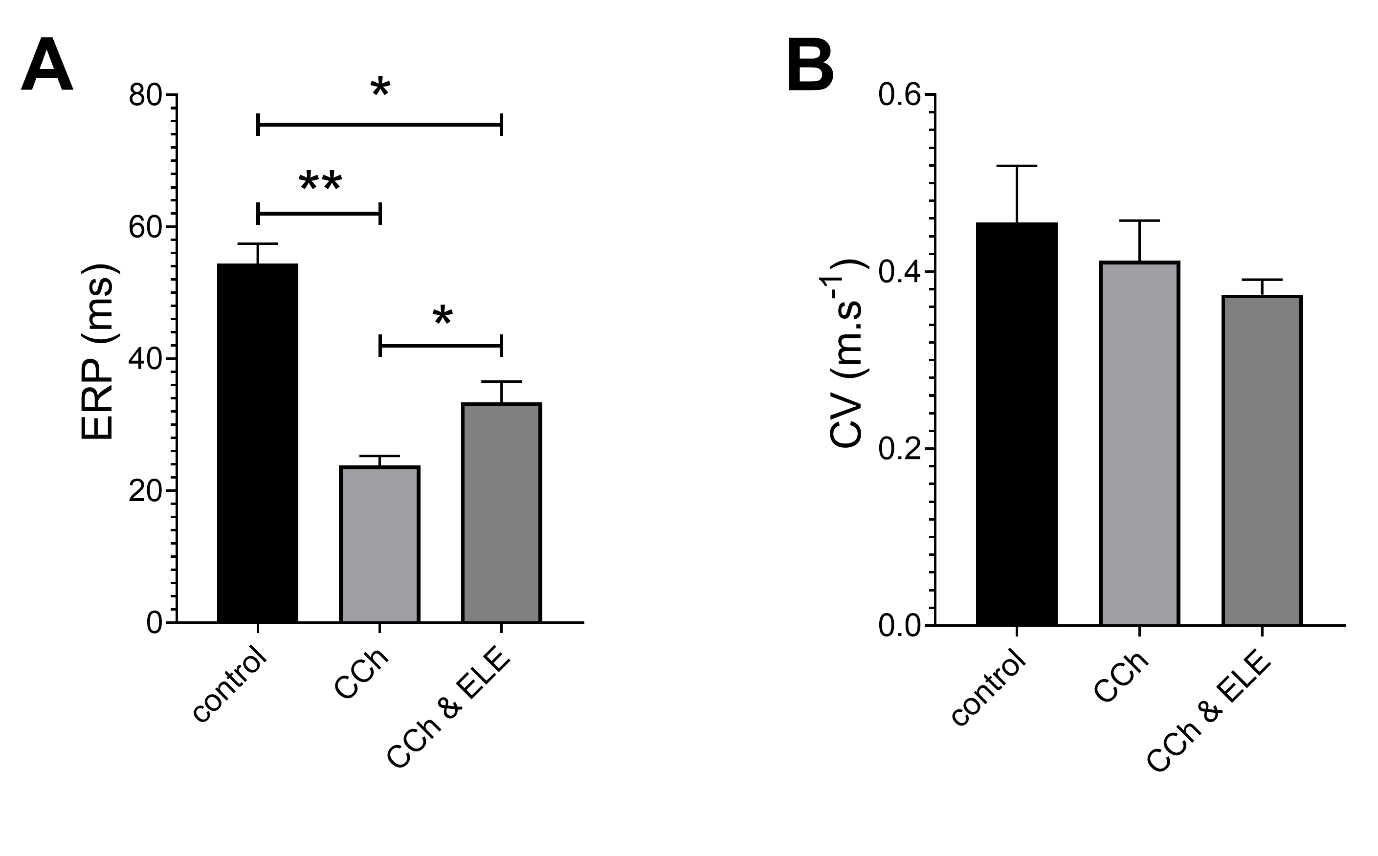


**Supplemental Figure 3**. The effect of eleclazine (ELE) on left atrial effective refractory period (ERP, **A**) and conduction velocity (CV, **B**) measured in Langendorff-perfused rat hearts in the presence of carbachol (CCh). Data are mean (±SEM). *, P<0.05, **, P<0.01; Tukey post-hoc test with repeated measures one-way ANOVA.


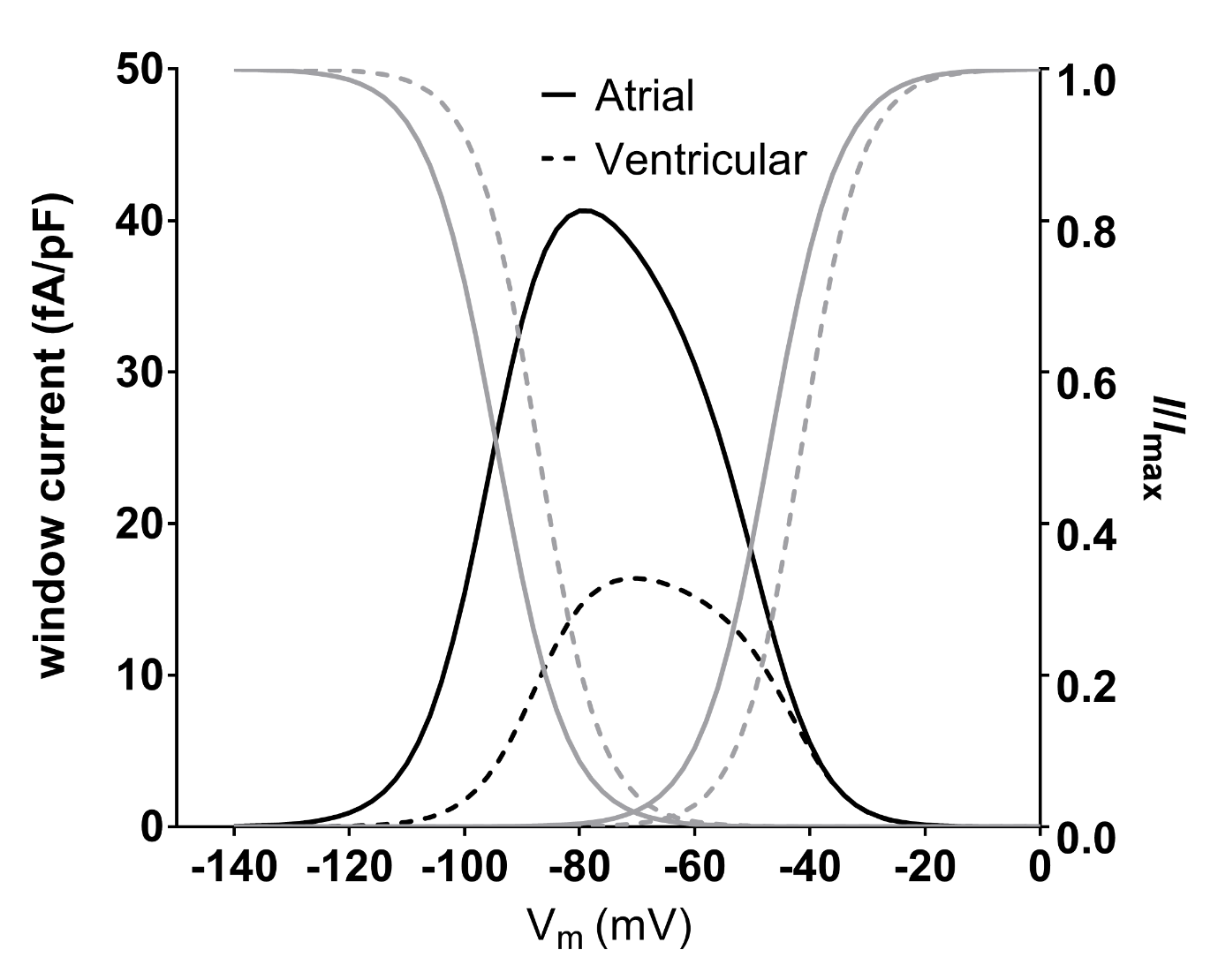


**Supplement Figure 4.** The window current in atrial and ventricular myocytes. Data plotted against the left-hand axis are window current densities calculated from the parameters presented in Supplemental Table 2 for atrial (solid black line) and ventricular (dashed black line) myocytes. Data plotted against the right-hand axis show steady-state voltage-dependent activation and inactivation curves (*I*/*I*_max_) according to the fitted parameters presented in Supplemental Table 2 for atrial (solid gray lines) and ventricular (dashed gray lines) myocytes.

**Supplemental Tables**

|  | **fast *I*_Na_** | | ***I*_NaL_** | |
| --- | --- | --- | --- | --- |
| **Constituent** | **Internal** | **External** | **Internal** | **External** |
| NaCl | 5 | 5 | 5 | 70 |
| CsCl | 20 | 140 | 20 | 75 |
| CsF | 110 | --- | 110 | --- |
| MgCl_2_ | 1 | 1 | 1 | 1 |
| CaCl_2_ | --- | 1 | --- | 1 |
| CoCl_2_ | --- | 1 | --- | 1 |
| D-glucose | --- | 10 | --- | 10 |
| EGTA | 5 | --- | --- | --- |
| BAPTA | --- | --- | 10 | --- |
| HEPES | 5 | 10 | 5 | 10 |
| pH | 7.2 | 7.3 | 7.2 | 7.3 |

**Supplemental Table 1**. Composition of solutions for recording of fast *I*_Na_ and *I*_NaL_ pH was adjusted using CsOH. Pipette solutions did not contain ATP, as has been reported previously for the recording of sodium currents^20-23^. Note that, for the recording of *I*_NaL_, BAPTA was used in place of EGTA as a Ca^2+^ chelator in the pipette solution as it was found that this improved the duration of recordings in the presence of ATX-II.

|  | Atrial | Ventricular |
| --- | --- | --- |
| **Activation** |  |  |
| Number of myocytes | 10 | 10 |
| *G_max_* (nS/pF) | 1.43 ± 0.14 | 1.33 ± 0.14 |
| *V_half,act_* (mV) | -47.0 ± 1.44 | -41.5 ± 1.32* |
| slope (mV) | 6.03 ± 0.86 | 5.25 ± 0.79 |
|  |  |  |
| **Inactivation** |  |  |
| Number of myocytes | 11 | 12 |
| *V_half,inact_* (mV) | -94.3 ± 0.46 | -87.2 ± 0.35**** |
| slope (mV) | -6.06 ± 0.40 | -5.44 ± 0.31 |

**Supplemental Table 2.** Fitted parameters of steady-state activation and inactivation of *I*_Na_ in atrial and ventricular myocytes. Data presented in Figure 1 and Figure 2 were fitted with *Supplemental Equations 1* and *4*, respectively. *, P<0.05; ****, P<0.0001; Student’s unpaired t-test.

|  | Atrial | Ventricular |
| --- | --- | --- |
| Total number of myocytes | 25 | 25 |
| *Log_10_IC_50_* (M) | -6.66 ± 0.025 | -6.75 ± 0.03 |
| *HillSlope* | -1.12 ± 0.075 | -0.88 ± 0.06 |
| IC_50_ (M) | 2.172×10^-7^ | 1.799×10^-7^ |

**Supplemental Table 3**. Parameters fitted to the concentration-dependence of *I*_NaL_ inhibition by ELE shown in Figure 3 using *Supplemental Equation 5*.

|  | Atrial (12) | | Ventricular (9) | |
| --- | --- | --- | --- | --- |
| DI (ms) | 110 | 40 | 110 | 40 |
| *Y_0_* | 1.02±0.06 | 1.07±0.06 | 1.09±0.10 | 1.16±0.10 |
| *b_ss_* | 0.75±0.004 | 0.53±0.01 | 0.86±0.01 | 0.64±0.01 |
| *K* (pulses^-1^) | 0.53±0.14 | 0.40±0.06 | 0.48±0.23 | 0.38±0.09 |

**Supplemental Table 4**. Parameters, with corresponding standard errors of fitting, fitted to the time course of use-dependent inhibition shown in Figure 4 according to *Supplemental Equation 7*. Numbers in parentheses indicate numbers of myocytes from which the data were obtained. The fitted data therefore had 477 (atrial) and 357 (ventricular) degrees of freedom. Rates of accumulation of ELE inhibition (*K*) were not significantly different by cell type (P=0.7817) or DI (P=0.4044) and there was no significant interaction between the two factors (P=0.9052, two-way ANOVA).

| **Source of variation** | Sum of Squares | DF | P value |
| --- | --- | --- | --- |
| time | 45.48 | 9 | <0.0001 |
| drug | 0.07706 | 1 | <0.0001 |
| cell type | 1.481 | 1 | <0.0001 |
| **interactions** | --- |  | --- |
| time × drug | 0.08104 | 9 | 0.0016 |
| time × cell type | 1.883 | 9 | <0.0001 |
| drug × cell type | 0.04391 | 1 | 0.0001 |
| time × drug × cell type | 0.0687 | 9 | 0.0069 |
| **Residual** | 0.7025 | 240 |  |

**Supplemental Table 5**. Summary of three-way analysis of variance of recovery from inactivation of atrial and ventricular myocytes under control conditions and in the presence of ELE (10 μM), as shown in Figure 7. DF represents degrees of freedom.

|  | Atrial | Ventricular | P |
| --- | --- | --- | --- |
| Total number of myocytes | 7 | 7 |  |
| *A_f_* | 0.35±0.04 | 0.27 ± 0.04 | 0.1429 |
| *τ_f_* (ms) | 2.51 ± 0.88 | 2.57 ± 1.25 | 0.9693 |
| *τ_s_* (ms) | 186±32 | 176±29 | 0.8208 |

**Supplemental Table 6**. Parameters fitted to the time course of recovery from ELE binding shown in *Figure 8*.

**References**

**1.** McMorn SO, Harrison SM, Zang W-J, Yu X-J, Boyett MR. A direct negative inotropic effect of acetylcholine on rat ventricular myocytes. Am J Physiol 1993;265:H1393-H1400.

**2.** Bryant S, Kimura TE, Kong CHT, Watson JJ, Chase A, Suleiman MS, James AF, Orchard CH. Stimulation of I_Ca_ by basal PKA activity is facilitated by caveolin-3 in cardiac ventricular myocytes. J Mol Cell Cardiol 2014;68:47-55.

**3.** Isenberg G, Klockner U. Calcium tolerant ventricular myocytes prepared by preincubation in a "KB medium". Pflugers Archiv 1982;395:6-18.

**4.** Tande PM, Bjornstad H, Yang T, Refsum H. Rate-dependent class III antiarrhythmic action, negative chronotropy, and positive inotropy of a novel Ik blocking drug, UK-68,798: potent in guinea pig but no effect in rat myocardium. J Cardiovasc Pharmacol 1990;16:401-410.

**5.** Zablocki JA, Elzein E, Li X, et al. Discovery of Dihydrobenzoxazepinone (GS-6615) Late Sodium Current Inhibitor (Late INai), a Phase II Agent with Demonstrated Preclinical Anti-Ischemic and Antiarrhythmic Properties. J Med Chem 2016;59:9005-9017.

**6.** Yuill KH, Convery MK, Dooley PC, Doggrell SA, Hancox JC. Effects of BDF 9198 on action potentials and ionic currents from guinea-pig isolated ventricular myocytes. Br J Pharmacol 2000;130:1753-1766.

**7.** Burnham KP, Anderson DR. Model selection and multimodel inference : a practical information-theoretic approach. 2nd ed. ed. New York: Springer; 2002.

**8.** Bergman C, Dubois JM, Rojas E, Rathmayer W. Decreased rate of sodium conductance inactivation in the node of Ranvier induced by a polypeptide toxin from sea anemone. Biochim Biophys Acta 1976;455:173-184.

**9.** Fuller H, Justo F, Nearing BD, Kahlig KM, Rajamani S, Belardinelli L, Verrier RL. Eleclazine, a new selective cardiac late sodium current inhibitor, confers concurrent protection against autonomically induced atrial premature beats, repolarization alternans and heterogeneity, and atrial fibrillation in an intact porcine model. Heart Rhythm 2016;13:1679-1686.

**10.** El-Bizri N, Xie C, Liu L, Limberis J, Krause M, Hirakawa R, Nguyen S, Tabuena DR, Belardinelli L, Kahlig KM. Eleclazine exhibits enhanced selectivity for long QT syndrome type 3–associated late Na^+^ current. Heart Rhythm 2018;15:277-286.

**11.** Rajamani S, Liu G, El-Bizri N, Guo D, Li C, Chen X-L, Kahlig KM, Mollova N, Elzein E, Zablocki J, Belardinelli L. The novel late Na^+^ current inhibitor, GS-6615 (eleclazine) and its anti-arrhythmic effects in rabbit isolated heart preparations. Br J Pharmacol 2016;173:3088-3098.

**12.** Rogers JC, Qu Y, Tanada TN, Scheuer T, Catterall WA. Molecular Determinants of High Affinity Binding of α-Scorpion Toxin and Sea Anemone Toxin in the S3-S4 Extracellular Loop in Domain IV of the Na+ Channel α Subunit. J Biol Chem 1996;271:15950-15962.

**13.** Zygmunt AC, Nesterenko VV, Rajamani S, Hu D, Barajas-Martinez H, Belardinelli L, Antzelevitch C. Mechanisms of atrial-selective block of Na^+^ channels by ranolazine: I. Experimental analysis of the use-dependent block. Am J Physiol 2011;301:H1606-H1614.

**14.** Caves RE, Cheng H, Choisy SC, Gadeberg HC, Bryant SM, Hancox JC, James AF. Atrial-ventricular differences in rabbit cardiac voltage-gated Na^+^ currents: basis for atrial-selective block by ranolazine. Heart Rhythm 2017;14:1657-1664.

**15.** Choisy SCM, Arberry LA, Hancox JC, James AF. Increased Susceptibility to Atrial Tachyarrhythmia in Spontaneously Hypertensive Rat Hearts. Hypertension 2007;49:498-505.

**16.** Kim S-J, Choisy SCM, Barman P, Zhang H, Hancox JC, Jones SA, James AF. Atrial Remodeling and the Substrate for Atrial Fibrillation in Rat Hearts with Elevated Afterload. Circ Arrhythm Electrophysiol 2011;4:761-769.

**17.** Kim S-J, Zhang H, Khaliulin I, Choisy SCM, Bond R, Lin H, El Haou S, Milnes JT, Hancox JC, Suleiman MS, James AF. Activation of Glibenclamide-Sensitive K_ATP_ Channels during *β*-Adrenergically-Induced Metabolic Stress Produces a Substrate for Atrial Tachyarrhythmia. Circ Arrhythm Electrophysiol 2012;5:1184-1192.

**18.** Allessie MA, Lammers WJ, Bonke IM, Hollen J. Intra-atrial reentry as a mechanism for atrial flutter induced by acetylcholine and rapid pacing in the dog. Circulation 1984;70:123-135.

**19.** Lammers WJ, Schalij MJ, Kirchhof CJ, Allessie MA. Quantification of spatial inhomogeneity in conduction and initiation of reentrant atrial arrhythmias. Am J Physiol 1990;259:H1254-H1263.

**20.** Josephson IR, Sperelakis N. Tetrodotoxin differentially blocks peak and steady-state sodium channel currents in early embryonic chick ventricular myocytes. Pflugers Archiv 1989;414:354-359.

**21.** Wang DW, Yazawa K, Makita N, George AL, Jr., Bennett PB. Pharmacological targeting of long QT mutant sodium channels. J Clin Invest 1997;99:1714-1720.

**22.** Zimmer T, Bollensdorff C, Haufe V, Birch-Hirschfeld E, Benndorf K. Mouse heart Na^+^ channels: primary structure and function of two isoforms and alternatively spliced variants. Am J Physiol 2002;282:H1007-H1017.

**23.** Camacho JA, Hensellek S, Rougier J-S, Blechschmidt S, Abriel H, Benndorf K, Zimmer T. Modulation of Na_v_1.5 Channel Function by an Alternatively Spliced Sequence in the DII/DIII Linker Region. J Biol Chem 2006;281:9498-9506.
